# Assessment and Antibiotic Resistance Profiling in *Vibrio* Species Isolated from Wild Birds Captured in *Danube Delta Biosphere* Reserve, Romania

**DOI:** 10.3390/antibiotics10030333

**Published:** 2021-03-22

**Authors:** Emöke Páll, Mihaela Niculae, Gheorghe F. Brudașcă, Rustam Kh. Ravilov, Carmen Dana Șandru, Constantin Cerbu, Diana Olah, Sergiu Zăblău, Adrian Valentin Potârniche, Marina Spinu, Gheorghiță Duca, Mariana Rusu, Magdalena Rzewuska, Aurel Vasiu

**Affiliations:** 1 Department of Infectious Diseases, Faculty of Veterinary Medicine, University of Agricultural Sciences and Veterinary Medicine, 400372 Cluj-Napoca, Romania; mihaela.niculae@usamvcluj.ro (M.N.); florinel.brudasca@usamvcluj.ro (G.F.B.); dana.sandru@usamvcluj.ro (C.D.Ș.); constantin.cerbu@usamvcluj.ro (C.C.); diana.olah@usamvcluj.ro (D.O.); sergiu-dan.zablau@usamvcluj.ro (S.Z.); adrian.potarniche@usamvcluj.ro (A.V.P.); aurel.vasiu@usamvcluj.ro (A.V.); 2 Subdivision of the Federal State Budgetary Institution of Science “Kazan Scientific Center of Russia Academy of Sciences”, Tatar Scientific Research Institute of Agriculture, 420088 Kazan, Russia; r.ravilov@kazanveterinary.ru; 3 Institute of Research and Development for Agro Montanology, 557085 Cristian, Sibiu, Romania; ghitaduca@yahoo.com (G.D.); icdmcristian@gmail.com (M.R.); 4 Instiute of Veterinary Medicine, Warsaw University of Life Sciences WULS, 02-786 Warsaw, Poland; magdalena_rzewuska@sggw.pl

**Keywords:** *Vibrio* spp., bacteria, wild birds, multidrug resistance, Danube Delta

## Abstract

Antimicrobial and multidrug-resistant bacteria are a major problem worldwide and, consequently, the surveillance of antibiotic-resistant bacteria and assessment of the dissemination routes are essential. We hypothesized that migratory birds, coming from various environments, would carry more numerous *Vibrio* strains than sedentary species, with increased risk to be passed to their contacts or environment in habitats they transit or nest in. Similarly, we presumed that strains from migratory birds will show multidrug resistance. A total of 170 oral and rectal swabs were collected from wild birds captured in different locations of the Danube Delta (Malic, Sfantu-Gheorghe, Letea Forest) and processed using standardized selective media. *V. cholerae* strains were confirmed by serology and molecular methods and, subsequently, their susceptibility was evaluated. The prevalence of *Vibrio* species by host species, habitat type, and location was interpreted. The isolated *Vibrio* species were identified as *Vibrio cholerae* 14.33%, *V. fluvialis* 13.33%*, V. alginolyticus* 12%*, V. mimicus* 17.33%, *V. vulnificus* 10.88%*,* with *V. parahaemolyticus* and *V. metschnikovii* (16%) also being prevalent. Of the 76 *Vibrio* spp. isolates, 18.42% were resistant towards at least three antimicrobials, and 81.57% demonstrated a multidrug resistance phenotype, including mainly penicillins, aminoglycosides, and macrolides. The results of the present study indicate higher numbers of *Vibrio* strains in migratory (74.66%) than in sedentary birds (25.33%), confirming our hypothesis. Furthermore, the increased pathogenicity of *Vibrio* spp. strains, isolated from wild migratory and sedentary birds, was confirmed by their increased multiple antibiotic resistance (MAR) index (0.09–0.81).

## 1. Introduction

The Danube is the second largest river in Europe, after the Volga, and also one of the most important rivers in Romania [1,2]. The Danube flows into the Black Sea through three branches: Chilia, Sulina, and Sfîntul Gheorghe, thus forming the Danube Delta. The Danube Delta is located in the northwestern part of the Black Sea basin and it is delineated by Sasik Lake, Midia Cape, and Ceatal Ismail (south) and by Chilia in the east [3]. In the Delta region, the Danube River splits into three main arms: Chilia (Kilia), Sulina, and Sf. Gheorghe (St. George) [4].

Letea Forest is the oldest natural reservation and protected area in the Danube Delta Biosphere Reserve. It is located between Sulina and Chilia branches of the Danube. It covers an area of approximately 2825 ha (6980 acres). On the other hand, the Sf. Gheorghe area represents a highly touristic region, with thousands of visitors coming in every year to enjoy the wonders of this peculiar location [5].

The Danube Delta is considered a paradise for birds, being visited annually by more than 330 species of wild migratory birds [6]. The Sfântu Gheorghe arm mouth [7] is an important starting point for the migration of Palearctic birds. Since the carriage of bacterial pathogens has been demonstrated in the case of several wild bird species, they may contribute to environmental contamination and transfer to humans [8,9], thus playing an important role in the epidemiology of human-associated zoonoses [10,11,12].

*Vibrio* species are natural residents of aquatic environments [13,14], characterized by high salinity and temperatures varying from 10 to 30 °C [15,16,17]. Numerous species *(V. cholerae*, *V. harveyi, V. parahaemolyticus, V. alginolyticus, V. anguillarum, V. splendidus*, *V. mimicus, V. vulnificus, V. hollisae,* and *V. fluvialis,* etc.) are responsible for severe infections in humans and animals [17,18,19]. Among these, *Vibrio cholerae* is the etiological agent for cholera, while *V. parahaemolyticus, V. mimicus, V. vulnificus, V. hollisae, V. furnissii, V. metschnikovii*, and *V. fluvialis* can cause acute gastroenteritis in humans following consumption of contaminated seafood [20,21,22,23], as well as *V. alginolytius* and *V. damsel*, which may be involved in wound and eye infection after exposure to sea water [18]. Another member of the genus, *V. harveyi*, may provoke mass mortality in marine invertebrates [24,25].

In addition to the many facets of their virulence, different species are characterized by antibiotic resistance. Drug-resistant strains of *V. cholerae* occur with increasing frequency [26,27,28]. Resistance genes to several antibiotics are located on large conjugative SXT elements that are integrated into *prfC* on the *V. cholerae* chromosome [27,28,29,30,31,32]. *V. cholerae* becomes drug-resistant by exporting drugs through efflux pumps, by chromosomal mutations, or by developing genetic resistance [33,34]. Streptomycin B resistance gene *strB* [27,33,35], sulfonamide resistance gene *sul2*, and trimethoprim resistance genes *dfrA1* and *dfr18* [27,36,37] also play a role in the acquisition of antibiotic resistance.

Such aspects are described for *Vibrio* species isolated from aquatic environments (surface water, sediment) and habitats from several regions [27,38,39,40] but no report characterizes the presence of *Vibrio* spp. strains isolated from wild birds captured in different locations of the Danube Delta, Romania. Therefore, we hypothesized that migratory birds, coming from various environments, would carry more numerous *Vibrio* strains than sedentary species, with increased risk to be passed to their contacts or environment in habitats they transit or nest in. Similarly, we presumed that strains from migratory birds will show multidrug resistance (MDR). The research carried out aimed at verifying these hypotheses.

## 2. Materials and Methods

**Figure 1:**
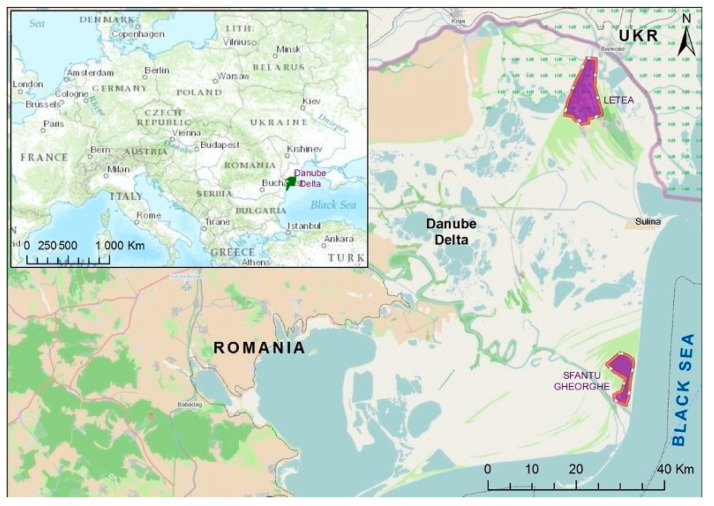
Sampling sites in the Danube Delta (Letea and Sfantu Georghe marked in purple, a total of 199 samples were collected from wild sedentary and migratory birds).

A total of 170 samples from wild birds were collected over a two-week period, during both spring and autumn, in Sfantu Gheorghe and Letea Forest of the Danube Delta (Figure 1).

The samples were collected from 44 migratory birds and 41 sedentary birds, such as the Eurasian sparrowhawk (*Accipiter nisus*), common chaffinch (*Fringilla coelebs*), great tit (*Parus major*), reed bunting (*Emberiza schoeniclus*), common snipe (*Gallinago gallinago*), wood sandpiper (*Tringa glareola*), black-crowned night heron (*Nycticorax nycticorax*), squacco heron (*Ardeola ralloides*), Eurasian hobby (*Falco subbuteo*), hooded crow (*Corvus corone cornix*), Eurasian teal (*Anas crecca*), common greenshank *(Tringa nebularia*)*,* Eurasian tree sparrow (*Passer montanus*), lesser whitethroat (*Sylvia curucca*), red-backed shrike (*Lanius collurio*), icterine warbler (*Hippolais icterina*), red-footed falcon (*Falco vespertinus*), garden warbler (*Sylvia borin*), common whitethroat (*Sylvia communis*), common kingfisher (*Alcedo atthis*), Eurasian blackcap (*Sylvia atricapilla*), barred warbler (*Sylvia nisoria*), black-and-white magpie (*Pica pica*), western jackdaw (*Corvus monedula*), Eurasian blue tit (*Parus caeruleus),* long tailed tit (*Aegithalos caudatus),* bearded reedling (*Panurus biarmicus),* hawfinch (*Coccothraustes coccothraustes*), and song thrush (*Turdus philomelos*), were sampled from both indicated areas. Both oral and cloacal swabs were collected individually from apparently healthy birds (*n* = 85) captured using mist nets according to standard practices. The birds were captured for scientific purposes (banding) by the Romanian Ornithological Centre (Institute of Biology, Romanian Academy of Sciences), were evaluated morphologically and physiologically, and were released upon the completion of the operation.

For initial bacterial isolation, the samples were inoculated in sterile alkaline peptone water (pre-enrichment) (APW, Oxoid) and incubated for 24 h at 30 °C. A total volume of 150 µL of enriched samples was added to thiosulphate–citrate–bile salts–sucrose agar (TCBS, Oxoid) and incubated for another 24 h for isolation of sucrose- and non-sucrose-fermenting *Vibrio* spp. detected based on the assessment of the cultural characteristics (green, yellow, bluish, and colorless colonies).

The strains presenting typical aspect (large yellow colonies, 2–3 mm diameter, colonies with blue to green centers, 3 mm diameter, large yellow mucoidal colonies, green colonies, 2–3 mm) were selected and cultured at 37 °C on trypticase soy agar (TSA, Oxoid) supplemented with 2% NaCl. All isolates were subjected to an oxidase test (Microbact) and species identification was carried out using the Rapid One NF Plus (ThermoFischer Scientific, Remel) system. Oxidase-positive isolates were further confirmed serologically by a slide agglutination test using *Vibrio cholerae* polyvalent agglutinating sera (Oxoid).

*Vibrio* spp. strains were subjected to PCR analysis using bacterial cell lysate as the source of template DNA. PCR primers for outer membrane protein (*ompW*) and regulatory protein (*toxR*) were used as described previously [41]. *The ompW* gene is involved in increasing the adaptability of virulent strains to different environmental conditions. The *toxR* gene is responsible for virulence through the production of specific proteins [42].

Bacterial DNA amplification was performed in a 25 µL reaction mixture containing 3 µL of template DNA (lysate) and 1 µL of each primer. The reaction mixture was subjected to amplification for 30 cycles, each of which consisted of three steps in the following order: denaturation of template DNA at 94 °C for 30 s, annealing of the template DNA at 64 °C for 30 s, and extension of the primers at 72 °C for 30 s. The amplified DNA fragments were separated on 1.5% agarose gel (Lonza) and visualized by staining with 5 µL RedSafe (Intron). One 100 bp DNA ladder (Bioline) was used as a DNA molecular weight standard.

The sensitivity patterns of the isolated *Vibrio* spp. strains were evaluated using the Kirby–Bauer disk diffusion technique on Mueller–Hinton agar (Oxoid) towards eleven antimicrobials: penicillin (P), 10 UI (Oxoid), erythromycin (E), 15 µg (Oxoid), ampicillin (AMP), 10 µg (Oxoid), chloramphenicol (C), 30 µg (Oxoid), amikacin (AK), 30 μg (Oxoid), kanamycin (K), 30 µg (Oxoid), oxytetracycline (OT), 30 µg (Oxoid), tetracycline (T), 25 mcg (Oxoid), enrofloxacin (ENF), 5 µg (KRKA), marbofloxacin (MAR), 5 µg (KRKA), and ciprofloxacin (CIP), 5 µg (Oxoid), belonging to six antimicrobial drug classes (tetracyclin, cloramphenicol, macrolides, penicillins, floroquinolones, aminoglycosides). The results were interpreted in accordance with CLSI 2020 guidelines. The multiple antibiotic resistance index was assessed based on the procedure described by Krumperman [43], calculating the MAR index as the number of antibiotics to which the isolate was resistant/total number of antibiotics against which the isolate was tested. Values lower than 0.2 were considered to represent low risk while those higher than 0.2 indicated a high risk [43]. Similarly, the MAR index for each antibiotic was calculated as the number of isolates resistant to the selected antibiotics, divided by the sum of the number of used antibiotics multiplied by the number of isolates [44]. Multidrug resistance (MDR) and extensive drug resistance (XDR) were considered as resistance to at least one agent in three or more antimicrobial categories or a lack of susceptibility to at least one agent in all but two or fewer antimicrobial categories, respectively, as according to Magiorakos and coworkers [45]. The results were analyzed with GraphPad Prism 5.00 software (GraphPad Software Inc., La Jolla, CA, USA) and Microsoft Excel. Average values were used to simplify the interpretation of the grouped data (GraphPad), while the graphical construal was supported by Excel.

## 3. Results

The present studies were conducted to assess the avian wildlife reservoir of *Vibrio* spp. in the Danube Delta and also evaluate their resistance to antibiotics.

The results of the microbiological assessment of the oral and cloacal samples are presented in Table 1.

**Figure 2:**
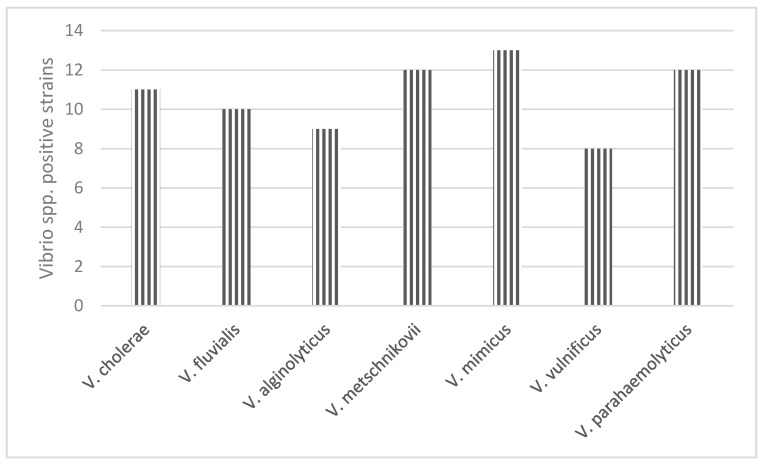
*Vibrio* spp. strains isolated from wild birds captured in Danube Delta Biosphere, Romania.

Based on the cultural characteristics and the results of the biochemical properties testing using the Rapid One NF plus system, seven species belonging to the genus *Vibrio* were detected and confirmed: *Vibrio vulnificus* 10.66% (*n* = 8), *Vibrio cholerae* 14.66% (*n* = 12), *Vibrio fluvialis* 13.33% (*n* = 10), *Vibrio parahaemolyticus* 16% (*n* = 12), *Vibrio mimicus* 17.33% (*n* = 13), *Vibrio metschnikovii* 16% (*n* = 12), and *Vibrio alginolyticus* 12% (*n* = 9) (Figure 2).

Most of these strains were isolated from samples collected from Eurasian hobby (*Falco subbuteo*), lesser whitethroat (*Sylvia curucca*), red-backed shrike (*Lanius collurio*), red-footed falcon (*Falco vespertinus*), common kingfisher (*Alcedo atthis*), hooded crow (*Corvus corone cornix*), and Eurasian blue tit (*Parus caeruleus*).

The majority (59.64%) of *Vibrio* spp. strains were isolated from migratory birds. Out of 75 samples, eleven (14.67%) isolates were positive in the PCR employing *ompW* and *toxR* primers. Nine of the DNA samples yielded a single amplicon of 588 bp in an *ompW*-based PCR assay and six samples yielded a 336 bp PCR product for *toxR*. About one third (36.36%) of the samples were positive for both *toxR* and *ompW* (Table 1).

**Figure 3:**
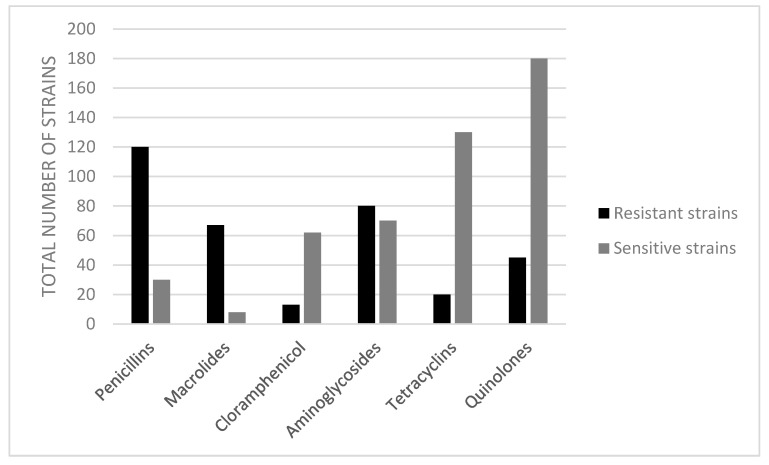
Incidence of antimicrobial resistance (represented by classes of antibiotics) in *Vibrio* spp. isolates from wild birds.

The isolated *Vibrio* spp. strains were evaluated for their level of antibiotic resistance. Based on the values of the inhibition diameters as compared against the CLSI guidelines 2020 in the Kirby–Bauer method, the bacterial strains were classified into resistant (R), intermediate (I), and susceptible (S) categories. The numbers of resistant strains in migratory and sedentary birds are indicated in Table 2. The percentage of total bacterial isolates demonstrating resistance or sensitivity to the 11 tested antibiotics is presented in Figure 3. In the present study, the resistance to erythromycin was the highest (88.16%), followed by penicillin (86.84%), amikacin (81.57%), and ampicillin (71.05%) and statistically significantly (*p* < 0.05) lower percentages were found for kanamycin (23.68%), quinolone class (enrofloxacine, marbofloxacine, and ciprofloxacine with 19.74%), and tetracycline class (tetracycline and oxitetracycline, both with 13.15%).

A MAR index value ≥0.2 was observed in 89.33% of the resistant pathogens. The MAR index calculated for isolated strains was 0.8 in 4 strains (all *V.cholerae* strains isolated from migratory birds *Ardeola ralloides, Turdus philomelos, Accipiter nisus),* 0.72 in 2 strains (*V. cholerae* strains isolated from *Falco subbuteo* and *Accipiter nisus*), 0.63 in 2 strains (*V. cholerae* strains isolated from *Falco subbuteo* and *Fringilla coelebs*), 0.54 in 4 strains, (*V. cholerae* isolated from *Gallinago gallinago, V. fluvialis* isolated from *Falco vespertinus, V. mimicus* from *Parus major*, and *Vibrio parahaemolyticus* isolated from *Sylvia borin*), 0.45 in 16 strains, 0.36 in 30 strains, 0.27 in 8 strains, 0.18 in 5 stains, and 0.09 in one strain (*V. fluvialis* associated with *Alcedo atthis*).

The isolates showed a high frequency of resistance to commonly used antibiotics, such as for each penicillin, erythromycin, and amikacin, between 90.66% and 92.00%, ampicillin (72%), and enrofloxacin (18.66%). Oxytetracycline, tetracycline, and ciprofloxacin were the most effective, with 86.66% of the strains being sensitive to oxytetracycline and tetracycline while 80% were sensitive to ciprofloxacin. The MAR indexes for antibiotics showed values ranging from 0.1 to 0.8 (Table 2) and the encountered resistance types are presented in Table 3. The result indicated the presence of MAR, MDR, and also XDR in the isolated *Vibrio* spp.

## 4. Discussion

In our study, we investigated the incidence of clinically important *Vibrio* spp. in wild birds captured in the Danube Delta Biosphere Reserve, Romania. *Vibrio* species are described as natural residents of aquatic environments [16,26], with species such as *Vibrio parahaemolyticus* and *Vibrio vulnificus* detected in the natural flora of estuarine and coastal marine environments worldwide [46,47], and also from the sea and brackish water and sediments of both tropical and temperate regions, as well as from a variety of seafood [17,46]. Seven clinically important *Vibrio* spp. were isolated, predominantly, *V. mimicus, V. parahaemolyticus*, and *V. metschnikovii*, followed by *V. cholerae*, *V. vulnificus*, *V. alginolyticus,* and *V. fluvialis.* Our results indicated a high frequency of all these *Vibrio* species in both wild migratory and sedentary birds.

The presence of these bacteria has medical relevance, as *V. parahaemolyticus*, *V. cholerae,* and *V. vulnificus* are the main species associated with seafood-borne infections, while *V. alginolyticus*, *V. mimicus,* and *Grimontia hollisae* (previously known as *V. hollisae*) have been sporadically isolated in disease outbreaks [48]. *Vibrio vulnificus* is one of the emerging food- and waterborne zoonotic bacteria, indigenous to estuarine waters and shellfish worldwide; it is involved in gastroenteritis and primary septicemia after consumption of contaminated oysters [49,50,51,52].

Toxigenic *Vibrio cholerae* (O1, O139) represent a major public health problem in many areas of the developing world [53,54,55]. The importance of wild birds as potential vectors of disease has received recent renewed empirical interest, especially regarding human health [9,23]. Besides other *Vibrio* spp., our results indicated the presence of *V. cholerae* in both sedentary and migratory birds, but with a higher frequency in migratory birds. Wild birds possess an important role in the epidemiology of *Vibrio* spp.-associated outbreaks [56,57] due to the particularities of their habitat and the interrelations with human activities (boat fishing, fish processing areas, and shellfish beds). Prolonged survival and persistence of *Vibrio* spp. in wild birds facilitate the possible contamination of the environment, especially in the case of migratory birds regarded as carriers for several bacterial pathogens, including several *Vibrio* species. Wild aquatic birds are a vehicle of *V. vulnificus* and *V. parahaemolyticus* in winter [58], so these bacteria can be potentially transported long distances [59]. Carrier status of *V. cholerae* in birds has a major implication for public health. The infection in birds occurs during their feeding in areas polluted by human or animal *V. cholerae* O1 carriers. Migratory birds may transport this pathogenic organism to different areas. Additionally, the transformation of non-O1 *V. cholerae* to an O1 serovar was demonstrated at the intestinal level of the birds [60].

The results of Nandi et al. (2000) [41] indicate that the *toxR* gene is involved in the regulation and expression of several genes of *V. cholerae*. The ToxR regulon (regulatory protein) is required in *Vibrio cholerae* for transcriptional activation of the *toxT* gene, which, in turn, activates numerous genes involved in the virulence of the bacterium [61,62,63]. Nevertheless, a low prevalence (7.9%) of this gene was found in the present study, indicating a potentially reduced overall virulence of the isolated strains, but not necessarily their decreased pathogenicity. Several studies indicate different degrees of resistance to antibiotics of isolated *Vibrio* spp. strains. The results of Li and coworkers [64] indicated a sensitivity of *Vibrio* species against streptomycin, rifampicin, kanamycin, tetracycline, and polymyxin B. These results could not be correlated with the studies of Okoh and Igbinosa (2010) [24] which reported percentages of resistance of 100, 90, 70, and 80 to trimethoprim, penicillin, cotrimoxazole, and streptomycin, respectively, in *Vibrio fluvialis*; 92, 82 90, and 100, respectively, to cephalothin in *V. vulnificus, V. parahaemolyticus, V fluvialis*, and *V*. *metschnikovii*, as well as resistance to ampicillin in all isolated *Vibrio* strains. Our findings were similar to those of Okoh and Igbinosa [24], especially in terms of strains resistant to penicillin and ampicillin. The authors isolated strains which exhibited various degrees of resistance toward tested drugs: tetracycline, marfloxin, and chloramphenicol (50%), penicillin, amikacin, and erythromycin 100%, ciprofloxacin (41.66%), oxytetracycline (58.33%), and ampicillin (83.33%), with six different resistance patterns being observed. You and coworkers (2016) [65] also isolated antibiotic-resistant and multidrug-resistant *Vibrio* spp. from aquatic environments, with high frequencies of resistance against erythromycin (81.8–95.7%), ampicillin (42–82%), and mecillinam (42–55%).

Similarly, the percentage of resistant strains was dependent on the antibiotic rather than on the class, and high resistance percentages indicated the differences in antimicrobial potency between the antibiotics in current human and veterinary use. Furthermore, the MAR values in the present study, as high as 0.2 to 0.8, supported the assumption that the isolated strains originated from a high-risk source of contamination and presented increased risk to public health. This hypothesis is further sustained by the presence of MDR and XDR in a large proportion (Table 3). Deng et al. (2020) [66] isolated *Vibrio* species, with a high prevalence of resistance to vancomycin, amoxicillin, midecamycin, and furazolidone, moderate prevalence of resistance to tobramycin, rifampicin, gentamicin, and tetracycline, and low prevalence of resistance to erythromycin, trimethoprim–sulfamethoxazole, doxycycline, and chloramphenicol. We found similar results for penicillins, but not for tetracyclines and erythromycin, were the results showed the opposite, with low and high prevalence of resistance, respectively.

Matyar et al. (2008) [67] showed multidrug resistance in Gram-negative bacteria isolated from aquatic environments and shrimp samples. Okoh and Igbinosa (2010) [24] identified multidrug-resistant non-cholera *Vibrio* spp. isolates showing resistance to all the antibiotics traditionally used to treat cholera. The study of Shivakumaraswamy et al. (2019) [68] revealed a high occurrence of antibiotic resistance in bacteria from animals and other natural environments.

Miyasaka and coworkers [59] evaluated the spatial and temporal distribution of *Vibrio* spp. in wild birds from Japan. The most important species of *Vibrio* were *Vibrio parahaemolyticus* and *Vibrio vulnificus*. The isolation of these species from feces demonstrated that the investigated avian species represent a vehicle for *V. parahaemolyticus* and *V. vulnificus* and also a habitat to survive during winter [59]. In this study, we isolated 12 strains of *V. parahaemolyticus* and eight strains of *V. vulnificus*, these strains mainly coming from migratory birds (*Tringa glareola, Nycticorax nycticorax, Sylvia curucca, Sylvia borin, Sylvia nisoria, Ardeola ralloides, Emberiza schoeniclus, Accipiter nisus, Anas crecca, Hippolais icterina*).

The emergence and spread of multidrug-resistant bacteria in natural environments represent a serious impact on animal and human health [69,70,71].

Wild birds have been not only postulated, but also demonstrated, as sentinels, reservoirs, and potential spreaders of antibiotic resistance [72,73,74,75,76]. Based on the results recorded by these authors, solid scientific evidence indicates the significant epidemiological role of wild birds in the dissemination of multidrug-resistant bacteria through migration.

Isolation of resistant bacterial strains from wilds birds highlights the potential hazard to both humans and animals given the transmission to humans and animals and vice versa [74,75,77]. The fecal shedding of the resistant strains allows environmental contamination.

However, most of these published studies are focused on *Escherichia coli* and/or *Enterococcus* spp. [73,78]. There is little information regarding the antimicrobial resistance level of *Vibrio* species isolated from wild birds, therefore, we believe that our study brings important additional information on the prevalence of *Vibrio* spp. in wild birds.

Antimicrobial resistance was determined for the *Vibrio cholerae* strains isolated from the Danube River in Slovakia [36]. All strains were susceptible only to three antimicrobials: chloramphenicol, rifampicin, and tetracycline, while resistance was displayed towards kanamycin and streptomycin [36]. Complementarily, our study evaluates antibiotic resistance to all isolated strains. Antimicrobial resistance is a growing problem worldwide and represents a major medical and public health problem [24,28,70]; a high prevalence of multidrug resistance indicates a serious need for antibiotic surveillance programs [79].

## 5. Conclusions

Our study confirmed the presence of MDR *Vibrio* spp. in migratory and sedentary wild birds captured from two different locations in the Danube Delta. Both migratory and sedentary birds from sampling sites were positive for a broad spectrum of potentially pathogenic *Vibrio* spp., indicating a great risk to public health. The present results indicate that wild-living birds may be a latent source for further microbial pollution of various habitats, including nesting places, and thus transfer it to offspring in the Danube Delta region. Further studies are intended to assess the antibiotic resistance determinants to underline the role of wild birds as a reservoir of multidrug-resistant *Vibrio* spp., including *V. cholerae.* Additional samples should be taken for a better assessment of carriage frequency.

## Figures and Tables

**Table 1:** *Vibrio* spp. isolated from cloacal and oral specimens collected (*n* = 170) from wild birds in Danube Delta Biosphere, Romania (samples harvested from migratory and sedentary birds, isolated and characterized bacterial strains isolated by microbiology and molecular biology techniques).

Species	Habitat Type	No. Captured Birds	Positive Samples for *Vibrio* spp.	Bacterial Isolate	PCR	
					*ompW*	*toxR*
Squacco heron (*Ardeola ralloides*)	Migratory	2	2	*V. cholerae*	+	+
				*V. alginolyticus*	*-*	*+*
				*V. parahaemolyticus*	*-*	*-*
Common greenshank *(Tringa nebularia)*	Migratory	2	2	*V. fluvialis*	*-*	*+*
				*V. alginolyticus*	*-*	*-*
Reed bunting (*Emberiza schoeniclus*)	Migratory	2	1	*V. metschnikovii*	-	-
				*V. fluvialis*	*-*	*-*
				*V. parahaemolyticus*	*-*	*-*
Song thrush (*Turdus philomelos*)	Migratory	2	1	*V. cholerae*	*+*	*-*
				*V. alginolyticus*	*-*	*-*
				*V. mimicus*	*-*	*-*
Eurasian hobby (*Falco subbuteo*)	Migratory	2	2	*V. cholerae*	*+*	*+*
				*V. fluvialis*	*-*	*-*
				*V. alginolyticus*	*-*	*-*
				*V. mimicus*	*-*	*-*
Wood sandpiper (*Tringa glareola*)	Migratory	2	2	*V. mimicus*	-	-
				*V. vulnificus*	*-*	*-*
Black-crowned night heron (*Nycticorax nycticorax*)	Migratory	2	2	*V. vulnificus*	-	-
				*V. parahaemolyticus*	*-*	*-*
Common snipe (*Gallinago gallinago*)	Migratory	3	2	*V. cholerae*	*+*	*+*
				*V. mimicus*	*-*	*-*
Eurasian sparrowhawk (*Accipiter nisus*)	Migratory	2	1	*V. cholerae*	*+*	-
				*V. parahaemolyticus*	*-*	*-*
Common chaffinch (*Fringilla coelebs*)	Migratory	2	2	*V. mimicus*	-	-
Eurasian teal (*Anas crecca*)	Migratory	2	1	*V. mimicus*	-	-
				*V. vulnificus*	*-*	*-*
					*-*	*-*
Eurasian tree sparrow (*Passer montanus*)	Sedentary	2	2	*V. cholerae*	*+*	*-*
				*V. fluvialis*	*-*	*-*
Lesser whitethroat (*Sylvia curucca*)	Migratory	1	1	*V. metschnikovii*	*-*	*-*
				*V. mimicus*	*-*	*-*
				*V. vulnificus*	*-*	*-*
Red-backed shrike (*Lanius collurio*)	Migratory	1	1	*V. fluvialis*	-	-
				*V. alginolyticus*	*-*	*-*
				*V. metschnikovii*	*-*	*-*
				*V. parahaemolyticus*	*-*	*-*
Icterine warbler (*Hippolais icterina*)	Migratory	3	2	*V. parahaemolyticus*	-	-
Red-footed falcon (*Falco vespertinus)*	Migratory	2	2	*V. cholerae*	*+*	*-*
				*V. fluvialis*	*-*	*-*
				*V. alginolyticus*	*-*	*-*
				*V. metschnikovii*	*-*	*-*
Garden warbler (*Sylvia borin*)	Migratory	4	3	*V. vulnificus*	-	-
				*V. parahaemolyticus*	-	-
Common whitethroat (*Sylvia communis*)	Migratory	5	4	*V. alginolyticus*	*-*	*-*
				*V. metschnikovii*	*-*	*-*
Common kingfisher (*Alcedo atthis*)	Migratory	1	1	*V. cholerae*	*-*	*-*
				*V. fluvialis*	*-*	*-*
				*V. metschnikovii*	*-*	*-*
				*V. mimicus*	*-*	*-*
Eurasian blackcap (*Sylvia atricapilla*)	Migratory	1	1	*V. parahaemolyticus*	-	-
Barred warbler (*Sylvia nisoria*)	Migratory	3	1	*V. metschnikovii*	*-*	*-*
				*V. vulnificus*	*-*	*-*
Black-and-white magpie (*Pica pica*)	Sedentary	5	2	*V. cholerae*	*+*	*+*
				*V. metschnikovii*	*-*	
				*V. parahaemolyticus*	*-*	*-*
Western jackdaw (*Corvus monedula*)	Sedentary	4	2	*V. cholerae*	*-*	*-*
				*V. fluvialis*	*-*	*-*
Hooded crow (*Corvus corone cornix*)	Sedentary	7	3	*V. metschnikovii*	-	-
				*V. mimicus*	*-*	*-*
				*V. vulnificus*	*-*	*-*
				*V. parahaemolyticus*	*-*	*-*
Eurasian tree sparrow (*Passer montanus*)	Sedentary	4	2	*V. cholerae*	*+*	*-*
				*V. fluvialis*	*-*	*-*
Eurasian blue tit (*Parus caeruleus*)	Sedentary	2	2	*V. metschnikovii*	-	-
				*V. mimicus*	*-*	*-*
				*V. vulnificus*	*-*	*-*
				*V. parahaemolyticus*	*-*	*-*
Long tailed tit (*Aegithalos caudatus*)	Sedentary	2	0	-	-	-
Bearded reedling (*Panurus biarmicus*)	Sedentary	2	1	*V. alginolyticus*	*-*	*-*
				*V. metschnikovii*	*-*	*-*
				*V. mimicus*	*-*	*-*
Common chaffinch (*Fringilla coelebs*)	Sedentary	3	2	*V. cholerae*	-	-
				*V. mimicus*	*-*	*-*
Hawfinch (*Coccothraustes coccothraustes*)	Sedentary	8	5	*V. alginolyticus*	*-*	*-*
				*V. metschnikovii*	*-*	*-*
				*V. mimicus*	*-*	*-*
Great tit (*Parus major*)	Sedentary	2	2	*V. fluvialis*	-	-
				*V. mimicus*	*-*	*-*

**Table 2:** Multiple antibiotic resistance (MAR) index of antibiotics against isolated *Vibrio* spp.

Antimicrobial Class	Drug	Total Number of Resistant Strains Isolated from Migratory Birds, by Antimicrobial	Total Number of Resistant Strains Isolated from Sedentary Birds, by Antimicrobial	MAR Index for the Tested Antibiotics
Penicillins	Penicillin	48	18	0.8
	Ampicillin	37	17	0.6
Macrolides	Erythromycin	47	20	0.8
Cloramphenicol	Chloramphenicol	12	1	0.1
Aminoglycosides	Amikacin	45	17	0.7
	Kanamycin	11	7	0.2
Tetracyclines	Oxytetracycline	10	0	0.1
	Tetracycline	8	2	0.1
Quinolones	Enrofloxacin	11	4	0.1
	Marbofloxacin	10	5	0.1
	Ciprofloxacin	9	6	0.1

**Table 3:** Resistance types observed in the isolated *Vibrio* spp.

Number of Antibiotic Classes to Which Isolated Strains Showed Resistance	Number/% of Resistant Strains	Resistance Type
1	1 (1.32)	-
2	5 (6.58)	MAR
3	35 (46.05)	MDR
4	21 (27.63)	XDR
5	10 (13.16)	XDR
6	4 (5.26)	XDR

MAR-multiple antibiotic resistance, MDR-multidrug resistance, XDR-extensively drug resistance.

## Data Availability

The data presented in this study are available on request from the corresponding author.
